# Whole-genome sequence of a high-temperature edible mushroom *Pleurotus giganteus* (zhudugu)

**DOI:** 10.3389/fmicb.2022.941889

**Published:** 2022-08-16

**Authors:** Hailong Yu, Meiyan Zhang, Yating Sun, Qiaozhen Li, Jianyu Liu, Chunyan Song, Xiaodong Shang, Qi Tan, Lujun Zhang, Hao Yu

**Affiliations:** ^1^National Engineering Research Center of Edible Fungi, Institute of Edible Fungi, Shanghai Academy of Agricultural Sciences, Shanghai, China; ^2^College of Horticulture, Shenyang Agricultural University, Shenyang, China; ^3^Shandong Provincial Key Laboratory of Applied Mycology, School of Life Sciences, Qingdao Agricultural University, Qingdao, China

**Keywords:** *Pleurotus giganteus*, genome, edible mushroom, white-rot fungi, mating locus, CAZymes

## Abstract

Most of the sequenced wood-rotting edible mushroom produce fruiting body at relatively low temperatures. Little information has been known about the high-temperature wood-rotting mushroom. Here, we performed *de novo* sequencing and assembly of the genome of a high-temperature edible mushroom *Pleurotus giganteus* from a monokaryotic strain zhudugu2 using the Illumina and Pac-Bio CLR sequencing technologies. *P. giganteus*, also known as Zhudugu in China, is a well-known culinary edible mushroom that has been widely distributed and cultivated in China, Southeast Asia, and South Asia. The genome consists of 40.00 Mb in 27 contigs with a contig N50 of 4.384 Mb. Phylogenetic analysis reveals that *P. giganteus* and other strains in *Pleurotus* clustered in one clade. Phylogenetic analysis and average nucleotide identity analysis indicated that the *P. giganteus* genome showed a closer relationship with other *Pleurotus* species. Chromosome collinearity analysis revealed a high level of collinearity between *P. ostreatus* and *P. giganteus*. There are 12,628 protein-coding genes annotated in this monoploid genome. A total of 481 enzymes accounting for 514 carbohydrate-active enzymes (CAZymes) terms were identified in the *P. giganteus* genome, including 15 laccases and 10 class II peroxidases predicted in the genome, which revealed the robustness of lignocellulose degradation capacity of *P. giganteus*. The mating-*A* type locus of *P. giganteus* consisted of a pair of homeodomain mating-type genes *HD1* and *HD2*. The mating-*B* type locus of *P. giganteus* consisted of at least four pheromone receptor genes and three pheromone genes. The genome is not only beneficial for the genome-assisted breeding of this mushroom but also helps us to understand the high-temperature tolerance of the edible mushroom.

## Introduction

*Pleurotus giganteus* is a culinary edible mushroom that has been recorded in tropical and subtropical regions, such as China, Malaysia, Sri Lanka, Indonesia, Vietnam, Laos, and Thailand, and has been commercially cultivated in recent years (Karunarathna et al., 2012; Phan et al., 2019). *P. giganteus* is known as “Zhudugu” in China due to the organoleptic properties that resemble pork stomach, and it is also named “Dabeisangu,” “Dabeixungu,” “Dalougougu,” “Dabeixianggu,” and “Sungu” in China because that the mature fruiting body of *P. giganteus* is like a goblet and funnel (Phan et al., 2019). *P. giganteus* is a wood-rotting fungus that uses sawdust and cottonseed hull as the main growth substrates. Soil casing methods are the major method for the fruiting production of this mushroom, either buried on the ground or the shelf in the factory. *P. giganteus* was also regarded as a multi-functional food supplement due to the high levels of nutrients and bioactive compounds in this species, such as polysaccharide, uridine, lipids, and feruloyl esterase, with neurite outgrowth, antioxidant, anti-candida, antitumor, hepatoprotective, and amylase inhibitory activities (Phan et al., 2012, 2013, 2014, 2015; Wang et al., 2014; Tian et al., 2016; Baskaran et al., 2017; Debnath et al., 2019).

By studying the mating system, Dong et al. (2010) Reported that *P. giganteus* has a typical tetrapolar heterothallic mating system. The distribution of the four mating types among spore monokaryons was ∼1:1:1:1 (Dong et al., 2010; Yu et al., 2021). Intraspesies et al. (2020) selected new hydrides of *P. giganteus* with high biological efficiency using intraspecific mating (Intraspesies et al., 2020; Li et al., 2021). Bioactive compounds and the mating system of this mushroom have been studied for many years; however, molecular and genetic studies on *P. giganteus* are rare due to the lack of genomic information. Owing to the advent of single-molecule real-time sequencing technologies, the continuity of mushroom genome assemblies [such as *Lentinus edodes* (Zhang et al., 2021; Yu et al., 2022), *Agrocybe cylindracea* (Liang et al., 2020), *Auricularia heimuer* (Yuan et al., 2019; Fang et al., 2020), *Stropharia rugosoannulata* (Li et al., 2022), *Russula griseocarnosa* (Yu et al., 2020), *Hericium erinaceus* (Gong et al., 2020), *Phellinus gilvus* (Huo et al., 2020), etc.] reached several orders of magnitude higher when compared with Illumina assemblies.

Here, we report a high-quality *de novo* genome assembly of *P. giganteus* through a combination of PacBio CLR and Illumina sequencing. Comparative analysis was conducted between the genome of *P. giganteus* and the other 23 published fungi genomes. Repeat sequences, carbohydrate-active enzymes (CAZymes), lignocellulose degradation enzymes, mating related genes were also analyzed. The *P. giganteu*s genome sequence will help understand the molecular mechanisms and evolution of this important edible mushroom.

## Materials and methods

### Strains and culture condition

The *P. giganteus* strain “Shen Xun 1 Hao” was selected for *de novo* genome sequencing and maintained in the Improved and Standardized Spawn Breeding Center (ISSBC), Shanghai Academy of Agricultural Sciences, China. The strain was domesticated from the wild strain collected in Fujian province in China and identified as a new mushroom variety by Shanghai Agricultural Technology Promotion Center. The *P. giganteus* strains “Shen Xun 1 Hao” were cultivated and maintained on potato dextrose agar (PDA) plates. For fruiting body production, strain Shen Xun 1 Hao was inoculated into the solid media [40% (w/w) sawdust, 40% (w/w) cottonseed hull, 18% (w/w) wheat bran, and 2% (w/w) gypsum powder] in the polypropylene bag. Vegetative growth of *P. giganteus* mycelia was carried out at 25°C with a humidity of 70-80%. After the mycelia occupied the full culture bag, the polypropylene bag was open and soil was covered on the top of the media. The fruiting body formed under the stimulation of temperature, water, and light. The monokaryotic strain zhudugu2 was obtained by selecting from the protoplasm of the strain “Shen Xun 1 Hao.”

### Genome sequencing

One monokaryon (zhudugu2) of the dikaryon strain “Shen Xun 1 Hao” was originally isolated by protoplast monokaryotization (Zhao and Chang, 1993). The obtained monokaryon zhudugu2 was cultured on 20 Potato Dextrose Agar (PDA) plates covered with cellophane at 25°C in darkness for 16 days. These mycelia were then collected, frozen in liquid nitrogen, and used for genome sequencing and chromosome-level genome construction. The genome of strain zhudugu2 was extracted using the NucleoBond HMW DNA kit (Macherey-Nagel, Düren, Germany). The concentration and quality of the DNA were analyzed using Thermal Nanodrop One (United States, CA) and Agilent 2100 Bioanalyzer System (United States, CA). The *P. giganteus* genome was sequenced using Illumina NovaSeq 6000 (paired-end, 2 × 150 bp, insert size, 400 bp) and PacBio Sequel sequencing platforms (CLR mode) by Personalbio Technology (Shanghai, China).

### Genome assembling and gene prediction

The PacBio reads were *de novo* assembled using Falcon and CANU software (Koren et al., 2017). Illumina sequencing reads were filtered using FastQC software. The assembled contigs were corrected using pilon v1.24 software Illumina short reads (Walker et al., 2014). The assembly completeness was evaluated with QUAST v5.1.0rc1 software (Gurevich et al., 2013) with the Illumina reads. The *ab initio* gene prediction was performed using Augustus v 3.03, glimmerHMM v 3.0.1, and GeneMark-ES v 4.35 software (Majoros et al., 2004; Stanke et al., 2006; Ter-Hovhannisyan et al., 2008). The predicted genes were integrated using EVidenceModeler v r2012-06-25 software (Haas et al., 2008). The completeness of the assembled genome was also evaluated using BUSCO v5.1.2 software with comparison to lineage dataset fungi_odb10 (creation date: 2020-09-10, number of BUSCO markers: 758) (Manni et al., 2021). Repeat sequence was analyzed using RepeatModeler and RepeatMasker software (Chen, 2004; Flynn et al., 2020). RepBase database was used to predict sequences similar to known repeat sequences. *Ab initio* structure prediction was performed using RepeatModeler software. RepeatMasker was used to make the prediction using the constructed repeat sequence library.

### Functional annotation

Functional annotations of the predicted protein-coding sequences (CDSs) were obtained using eggNGOmapper software (Cantalapiedra et al., 2021). Pfam and SwissProt function annotation was performed by sequence alignment against Pfam-A database (database version: Pfam35.0) and SwissProt database (2022-04-30) by Hmmer 3.3.2 and diamond 0.9.21, respectively.

### Comparative genomic analysis

The pairwise average nucleotide identity (ANI) values between genomes were analyzed using FastANI software (Jain et al., 2018). Collinearity analysis was performed by MCScanX-jcvi software based on the protein sequence from the GFF3 files of *P. giganteus*, *P. ostreatus*, and *Agaricus bisporus* (Wang et al., 2012). Gene families and single-copy orthologous genes were analyzed using OrthoFinder v2.5.4 software (Emms and Kelly, 2019). The species tree was constructed using concatenate single-copy orthologous genes and visualized using FastTree software (Price et al., 2009).

### Identification of CAZymes

Annotation of CAZymes for the genome of *P. giganteus* was performed using dbcan version v3.0.2 software (Cantarel et al., 2009; Zhang et al., 2018). The database was downloaded from the dbCAN meta server^1^ (version of the database is V10). Hmmer software was used for the annotation of proteins with default parameters (HMMER E-Value < 1e-15, HMMER Coverage > 0.35).

### Identification of the mating locus

The mating-type locus of *P. giganteus* was analyzed using sequence alignment with diamond software (Buchfink et al., 2015). Mating type genes in other *Pleurotus* strains as the reference sequences. The genome of *P. eryngii* ATCC90797 was downloaded from the MycoCosm portal of the Joint Genome Institution (JGI). The *A* mating type locus of strain ATCC90797 is in scaffold1 between 196670 and 871073, and the *B* mating type locus is in scaffold49 between 196670 and 254852. The genome of *P. ostreatus* strain PC9 was also downloaded from the JGI database. The *A* mating type locus of strain PC9 is in scaffold4 between 2032971 and 2039163. Gene cluster structure was visualized using integrative genomics viewer software (Thorvaldsdóttir et al., 2013).

## Results and discussion

### Cultivation of *Pleurotus giganteus* and monokaryotic strain isolation

**FIGURE 1:**
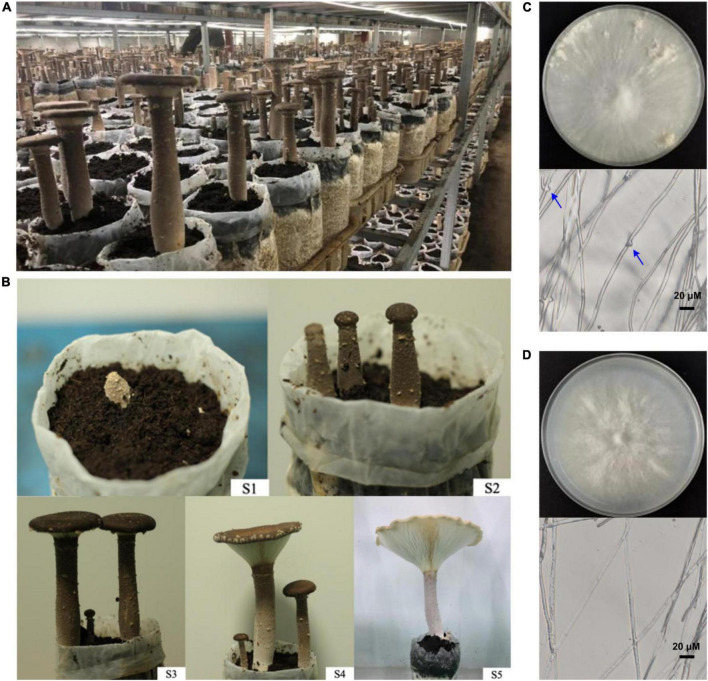
Life cycle of *P. giganteus*. **(A)** Industrially cultivated *P. giganteus*. **(B)** Fruiting body of *P. giganteus* at different stage of life cycle. **(C)** Dikaryotic mycelia of the *P. giganteus* Shen Xun 1 Hao strain. Clamp connections were indicated by arrows. **(D)** Monokaryotic mycelia of the *P. giganteus* zhudugu2 strain.

Figure 1 showed the cultivation situation and the main process of fruiting body development of *P. giganteus* (Shanghai, China, by our lab). At the S1 primordium stage, mycelium kinked to form fruiting body. The S2 growing stage showed the continued growth period after primordium formation. S3 phase is the elongation stage and the fruiting bodies continue to grow until the funnel is formed at this stage. S4 is the harvest stage when the fruiting body grew to 70–80% maturity and the cap is funnel-shaped. At mature stage S5, the fruiting body was fully mature and the cap was in the shape of a large funnel. The monokaryotic zhudugu2 strain was isolated from the protoplasm of the “Shen Xun 1 Hao” strain (Figure 1C). Figures 1C,D show the mycelia of the dikaryotic and monokaryotic mycelia of *P. giganteus*, respectively, and no clamp connection was observed on monokaryotic mycelia (Figure 1D).

### Genome assembly and annotation of *Pleurotus giganteus*

**FIGURE 2:**
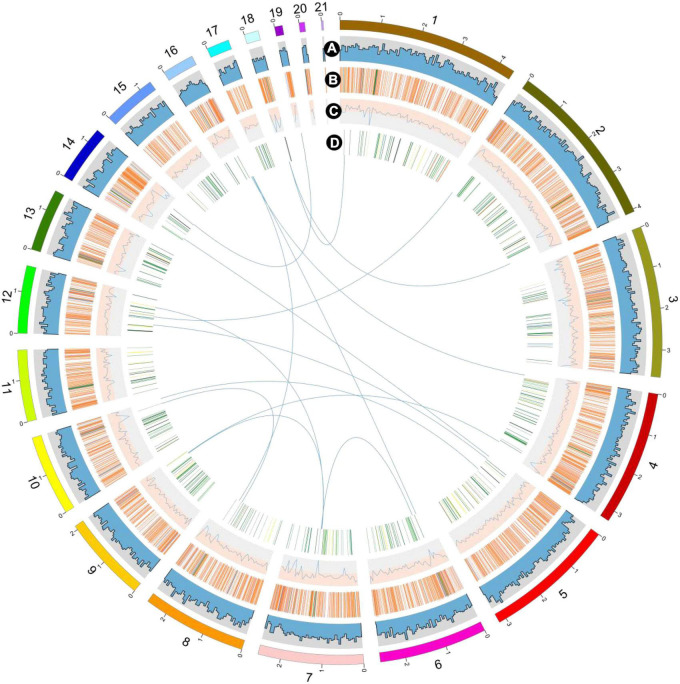
Overview of the *P. giganteus* genome assembly. The outermost layer of colored blocks is a circular representation of the 21 contigs with coding sequences, with a scale mark labeling each 1 Mb. **(A)** Gene density per window. **(B)** Repeat sequence density per window. **(C)** GC content per window. **(D)** carbohydrate-active enzymes (CAZymes). Links within and between chromosomes represent collinear blocks generated from MCScanX and JCVI software. For tracks **(A–C)**, the window size is 50 kb. The plot was visualized using Circos software.

The genome of the *P. giganteus* zhudugu2 strain was sequenced using PacBio and Illumina sequencing platforms. A total of 36,251,924 clean reads (∼130 ×) were obtained from Illumina sequencing which was used for k-mer analysis and genome polish. GenomeScope was used to generate a histogram of the depth distribution of the sequencing (*k* = 19) (Supplementary Figure 1). A single k-mer coverage peak was observed and the heterozygous rate was 0.02%. The results confirmed that strain zhudugu2 was monokaryotic. The approximately 877,248 million clean Nanopore reads (∼200 ×) were *de novo* assembled into 27 contigs with an N50 of 2.61 Mbp and an N90 of 1.36 Kbp. The total sequence length was 40,035,591 bp (Table 1) and the length of the largest contig is 4.38 Mbp (Figure 2). The GC content of *P. giganteus* genome was 50.5%. The integrity of the genome was evaluated using QUAST v5.1.0 software and determined to be 98.2%. The size of the *P. giganteus* genome is similar to those of other species in the *Pleurotus* genus. It a little larger than those of *Pleurotus tuber-regium*, *Pleurotus ostreatus* and *Pleurotus citrinopileatus*, but less than the genome size of *Pleurotus eryngii* and *Pleurotus tuoliensis*. The GC content of *P. giganteus* is similar to most of the *Pleurotus* species but larger than those of the *P. tuber-regium* strains.

**TABLE 1:** *De novo* genome assembly and features of *P. giganteus*.

Characteristics	*P. giganteus* zhudugu
Genome assembly size (Mb)	40.0
Scaffolds	27
Contigs	27
Longest Scaffold (kb)	4384
Scaffold N50 (kb)	2611
Scaffold N90 (kb)	1360
GC%	50.5%
Sequencing platform	PacBio CLR, Illumina

Repeat sequence was identified using RepeatMasker based on homology alignment and *ab initio* prediction and accounted for 11.36% of the *P. giganteus* genome (Figure 2B). The majority of repetitive sequences were LTR retrotransposons (5.01%), where 0.17% and 0.52% of the repeat element types were DNA transposons and simple repeats (Table 2). However, no LINEs and Satellites were predicted in the *P. giganteus* genome.

**TABLE 2:** Repeat element analysis in the *Pleurotus giganteus* genome.

Repeat elements	Copies (numbers)	Repeat size (bp)	Percentage of the assembled genome
LTR/Copia	376	448263	1.12%
LTR/Gypsy	675	1269506	3.17%
LTR/others	468	289672	0.72%
DNA transposons	49	66293	0.17%
Simple repeats	4983	206553	0.52%
Low complexity	749	40049	0.10%
Rolling-circles	44	101011	0.25%
Unclassified	4423	2126869	5.31%
total	11767	4548216	11.36%

A total of 12,628 gene models were predicted from the genome of *P. giganteus* with an average sequence length of 1761 bp. The concatenated length of CDSs was 2.22 Mbp, which accounted for 55.5% of the total genome (Supplementary Tables 1, 2). Of the identified genes, 9801 (77.6%) and 5494 (43.5%) genes were annotated by the EggNOG database and SwissProt database, respectively. Based on the similarity of protein domains, 7802 (61.8%) genes were annotated by the Pfam database. The completeness of *P. giganteus* genome assembly and gene prediction was also evaluated using the BUSCO software with fungi_odb10. The completeness of the zhudugu2 genome was 94.2% (Supplementary Figure 2). These results and the assembly parameters indicated that we generated a high-quality genome of *P. giganteus*.

### Comparative genomic analysis

**FIGURE 3:**
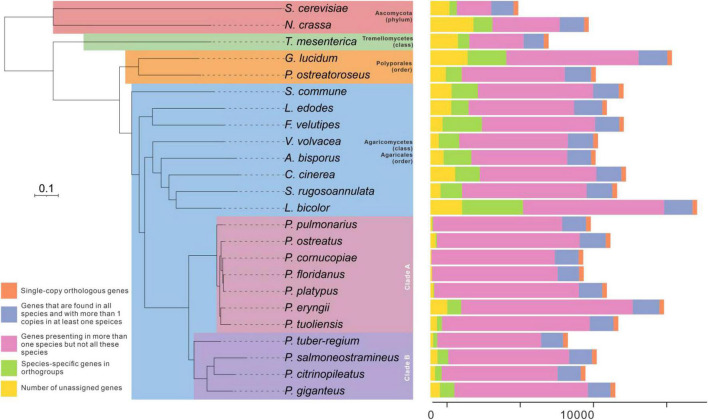
Comparison of genomes between *P. giganteus* with other 23 fungal species (21 Basidiomycetes and 2 Ascomycetes). The evolutionary relationship analysis was constructed based on 312 single-copy orthologous gene. Tree scale = 0.2.

*Pleurotus giganteus* was originally described as *Lentinus giganteus* and *Panus giganteus* due to its different fruiting body shapes at different stages in the life cycle (Samantha et al., 2016; Phan et al., 2019). Karunarathna et al. (2012) have corrected the classification of this strain to the *Pleurotus* genus based on ITS sequences (Samantha et al., 2016). To confirm the evolutionary relationship of *P. giganteus*, a comparative analysis of the *P. giganteus* genome and 23 fully sequenced fungi genomes (21 Basidiomycetes and 2 Ascomycetes) was performed. A phylogenetic tree constructed based on conserved single-copy orthologous gene alignment showed that *P. giganteus* had a close evolutionary relationship with other *Pleurotus* species (Figure 3). The strains in *Pleurotus* species were clustered into two main clades. *P. giganteus*, *P. tuber-regium*, *P. citrinopileatus*, and *P. salmoneostramineus* form one clade, while the other seven species formed the other clear clade. *P. giganteus* showed a closer evolutionary relationship with *P. tuber-regium* and *P. citrinopileatus.* Indeed, the fruiting bodies of the three species shared similar characteristics. They all have a complete circle cap with a dent in the middle, which look like a funnel. However, the fruiting body of the species on the other clade has a round cap on one side and a notch on the other side.

**FIGURE 4:**
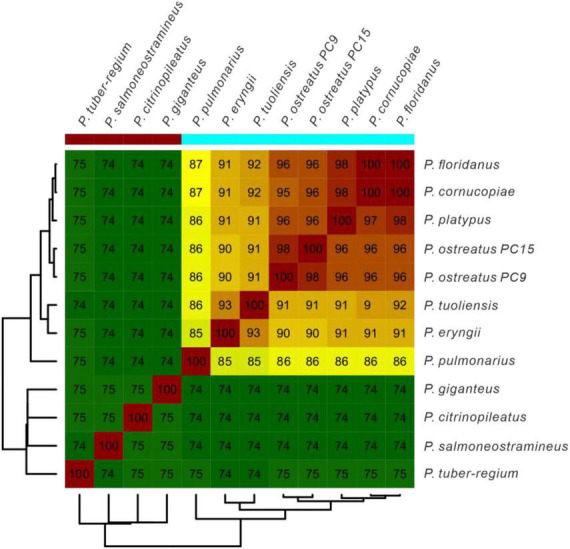
The average nucleotide identity (ANI) values based on the fastANI algorithm generated matrix for *Pleurotus genomes*. The clustering was constructed using Euclidean distance matrix.

Average Nucleotide Identity (ANI) analysis is a high-resolution taxonomic analysis method. To further confirm the evolutionary relationship, ANI analysis was performed to estimate genomic differences and relatedness between *Pleurotus* strains’ genomes. As a result in Figure 4, *Pleurotus* species in Clade B in the species tree showed lower genomic similarities (74 to 75%) with each other and *Pleurotus* species in Clade A. Whereas species in Clade A showed high genomic similarities between each other (85 to 100%). In a word, the results above confirmed that *P. giganteus* belong to the genus of *Pleurotus* according to the current classification.

To explore the genetic factor for the fruiting body shape and thermo-adaptation of P. giganteus, orthogroups of five commercially produced *Pleurotus* species were analyzed (Supplementary Table 4). As shown in Supplementary Figure 3 *P. giganteus* and *P. tuber-regium* shared 142 orthogroups that were missed in the other three species. Both *P. giganteus* and *P. tuber-regium* have a cup-shaped fruiting body and are distributed in the tropical zone. Therefore, these 142 orthogroups might related with fruiting body shape and thermo-adaptation. The 142 orthogroups contain 353 genes in *P. giganteus* genome, and 164 were annotated by the Pfam database (Supplementary Table 5). According to the Pfam annotation, most of these 164 genes were related to regulation, transposon, and protein digestion. For example, 43 genes contained F-box-like domain, which was first characterized as components of SCF ubiquitin-ligase complexes (Kipreos and Pagano, 2000). Ubiquitin was related with many important biological processes including thermotolerance (Panek et al., 2020; Zhang et al., 2022). The process of ubiquitination plays a key role in plants’ thermotolerance by eliminating denatured proteins (Zhang et al., 2022). In our previous study, proteins related to the ubiquitin-dependent protein catabolic process were enriched in heat-shock-induced proteins (Xu et al., 2021), which also indicates that ubiquitination played a key role in thermotolerance in mushroom. Therefore, regulation of gene expression, protein expression, and protein degradation might one of the important reasons for the characteristics of fruiting body shape and thermo-adaptation of *P. giganteus*. A detailed molecular mechanism needs to be investigated through differential expression analysis of genes or proteins in these species.

**FIGURE 5:**
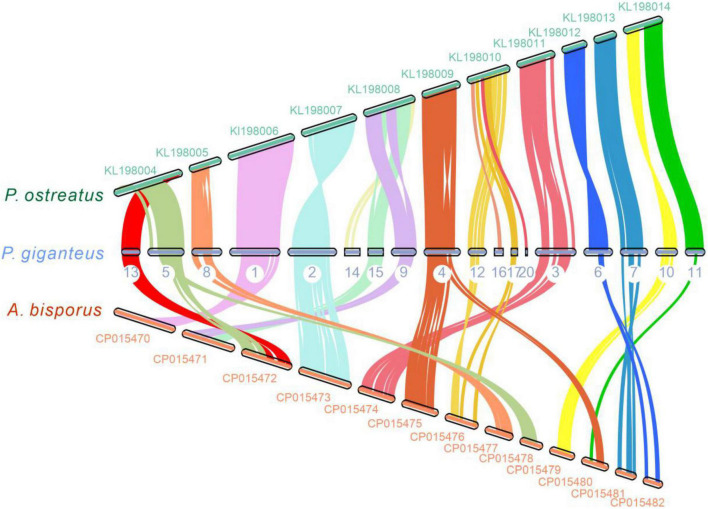
The genome collinearity among *P. giganteus*, *P. ostreatus*, and *Agaricus bisporus*. Each line connects a pair of collinearity blocks between two genomes. Different colors represent different scaffolds of *P. giganteus*. Scaffold with no collinearity was not shown.

Chromosome collinearity analysis of *P. giganteus* and other two edible mushrooms, which have chromosome levels of the genome, was performed using JCVI and MCScanX software (Figure 5). The results revealed high levels of collinearity between *P. ostreatus* and *P. giganteus*. According to the collinearity results, contig 5 and contig 13 may belong to the same chromosome, contig 9, 14, and 15 may belong to the same chromosome, and contig 12, 16, 17, and 20 may belong to the same chromosome. The connection of these contigs could be further confirmed using PCR or Hi-C technology (Teh et al., 2017). Rupture and fusion events were identified in contig 5, contig 8, contig 15, contig 9, contig 3 of *P. giganteus* compared to chromosomes from *A. bisporus*. More rupture and fusion events occurred between *P. giganteus* and *A. bisporus* than those between *P. ostreatus* and *P. giganteus*, which is in accordance with the phylogenetic analysis results.

### CAZymes in *Pleurotus giganteus* genome

**FIGURE 6:**
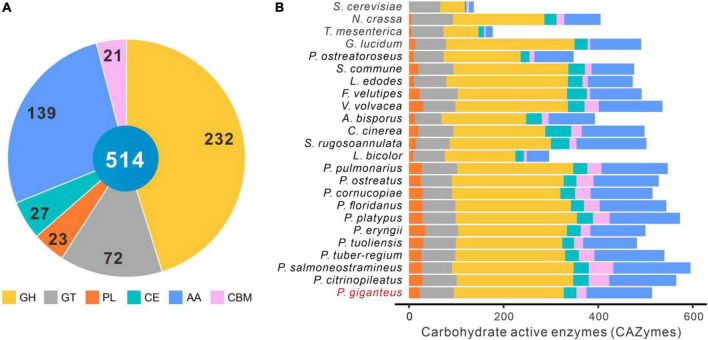
Carbohydrate-active enzymes (CAZymes) in *P. giganteus* and other 23 fungi. **(A)** The distribution of CAZymes categories in *P. giganteus*. **(B)** The distribution of CAZymes in other 23 fungi. The strain names of these fungi are the same as those in Figure 3. GH, glycoside hydrolase; GT, glycosyltransferase; PL, polysaccharide lyase; CE, carbohydrate esterase; CBM, carbohydrate-binding module; AA, auxiliary activity.

CAZymes are one of the most important gene families in the fungal genome, which are responsible for lignocellulose degradation and many other biological processes, such as development and stress response (Garron and Henrissat, 2019; Pallister et al., 2020). A total of 481 enzymes accounting for 514 CAZymes terms were identified in *the P. giganteus* genome with Hmmer software, including 232 GHs, 72 GTs, 23 PLs, 27 CEs, 139 AAs, and 21 CBMs (Figure 6A and Supplementary Table 3). The CAZymes of other 23 fungal species were analyzed using the same parameters with dbCAN2 database. As shown in Figure 6B, the number of CAZymes in *P. giganteus* is a little less than those in other *Pleurotus* species, except for *P. eryngii* and *P. tuoliensis*. The different CAZymes number might be caused by gene prediction methods, structural variation of genome, and alignment parameters. It can be sure that *P. giganteus* also has a powerful lignocellulose degradation capacity just like other members of *Pleurotus* genus. The results are consistent with the production processed, that we can use different lignocellulosic biomass to produce *P. giganteus* fruiting body.

*Pleurotus giganteus* had 139 AAs. Proteins in the AA category were mainly distributed in AA3, AA9, AA7, AA5, AA1, and AA2 families (Supplementary Table 3). Proteins in AA3 and AA9 categories were related to the degradation of cellumose and hemicellulose. The lignin-degrading enzymes, such as laccases and manganese peroxidases, were in the AA1 and AA2 categories. A total of 15 laccases and 10 class II peroxidases were annotated in the genome of *P. giganteus* with SwissProt database. These enzymes play key roles in the degradation of plant cell wall components (Manavalan et al., 2015; Suryadi et al., 2022). Therefore, the results indicated a robustness lignocellulose degradation of this strain, which is in consistent with the woody materials used in the fruiting body production.

### Identification of the mating locus

**FIGURE 7:**
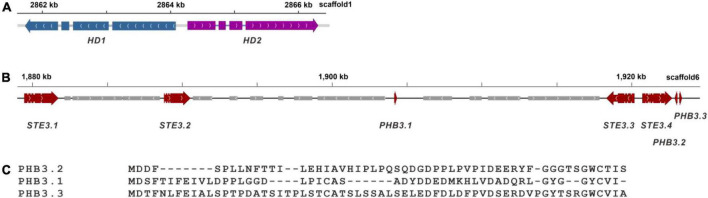
Gene structure of the mating type locus of *P. giganteus*. **(A)** Structure of A mating type locus in *P. giganteus*. **(B)** Structure of B mating type locus in *P. giganteus* zhudugu2**. (C)** Comparison of the mating pheromones predicted in *P. giganteus*.

Mating-type genes from *P. ostreatus* PC9 and *P. eryngii* ATCC 90797 were used as query sequences for the alignment to identify mating type locus in strain zhudugu2. The A mating type locus of *P. giganteus* was on scaffold 1. The total length of the locus was over 4 kb, consisting of two homeodomain genes (HD) of similar sizes. The two HD genes were transcribed in opposite directions as in *P. ostreatus* (Figure 7A) (Ju et al., 2020). Different from *P. ostreatus* PC9 and *P. giganteus* zhudugu2, *P. eryngii* ATCC 90797 has one *HD1* gene and one *HD2* gene transcribed in opposite directions and one extra HD2 gene is located on the other side of HD1. The amino acid sequence showed 52.7 and 51.1% sequence similarity with the HD1 in *P. ostreatus* PC9 and *P. eryngii* ATCC 90797, respectively. The HD2 showed 57.4 and 50.0% (49.5%) sequence similarity with those in strain PC9 and ATCC 90797, respectively.

Four pheromone receptor genes (*STE3.1*-*STE3.4*) and 3 pheromone genes (PHB3.1-PHB3.3) were predicted and clustered in a ∼45 kb gene cluster (Figures 7B,C). TMHMM prediction showed that all four pheromone receptors contained 7 trans-membrane domains. The gene organization of the *B* mating locus is different from that in *P. eryngii* (Ju et al., 2020) and *Pleurotus djamor* (James et al., 2004), which indicated the *B* mating type locus has evolved through multiple events of genome rearrangement like in other mushroom such as in *Flammulina velutipes* (Wang et al., 2016) and *Lentinula edodes* (Ha et al., 2019). The information provided here is important for the development of molecular markers for crossbreeding of *P. giganteus*.

## Conclusion

In summary, this is the first report of the whole genome of *P. giganteus*. Integrity, completeness, and collinearity analysis revealed the high quality of genome assembly. Comparative genome analysis confirmed that *P. giganteus* should be classified as *Pleurotus* genus instead of *Lentinus* and *Panus*. Identification of CAZymes revealed that *P. giganteus* had robustness lignocellulose degradation capacity. Repeat sequence analysis and mating locus could be used for the development of molecular markers in strain identification and breeding. The *P. giganteus* genome provided insights for basic research on the cultivation, nutrition, and medicinal utility of this mushroom.

## Data availability statement

The datasets presented in this study can be found in online repositories. The names of the repository/repositories and accession number(s) can be found below: https://ngdc.cncb.ac.cn/gsa, CRA006857.

## Author contributions

HLY, LZ, and HY conceived and designed the project and wrote the manuscript. HLY, MZ, YS, QL, JL, and CS performed the experiments. QT and HLY contributed reagents and materials. HLY, MZ, XS, and HY analyzed the data. All authors have read and approved the manuscript.

## Footnotes

1
http://bcb.unl.edu/dbCAN2/
