# Supplementary material for: Whole-genome sequence of a high-temperature edible mushroom Pleurotus giganteus (zhudugu)
Source: Front Microbiol. 2022 Aug 16;13:941889. doi: 10.3389/fmicb.2022.941889 (PMC9424821; doi:10.3389/fmicb.2022.941889)
Supplement: Supplementary file 1 [file Data_Sheet_1.pdf]

Whole Genome Sequence of an High Temperature Edible Mushroom *Pleurotus giganteus* (Zhudugu)

Hailong Yu[1†], Meiyan Zhang[1†], Yating Sun[1,2†], Qiaozhen Li[1],Jianyu Liu[1], Chunyan Song[1], Xiaodong Shang[1], Qi Tan[1], Lujun Zhang[1]* and Hao Yu[3]*

[1]National Engineering Research Center of Edible Fungi, institute of Edible Fungi, Shanghai Academy of Agricultural Sciences, Shanghai, China, [2]College of Horticulture, Shenyang Agricultural University, Shenyang, Liaoning, China, [3]Shandong Provincial Key Laboratory of Applied Mycology, School of Life Sciences, Qingdao Agricultural University, Qingdao, Shandong, China

* Correspondence: zhanglujun@saas.sh.cn (LJ.Z.); yuhao@qau.edu.cn (H.Y.) Tel: +86-532-5895-7640 (H.Y.)

† These authors contributed equally to this work

Mailing address: 700 Changcheng Road, Chengyang District, Qingdao, Shandong Province, People's Republic of China

Keywords: *Pleurotus giganteus*; genome; edible mushroom; white-rot fungi; mating locus, CAZymes

Figure S1

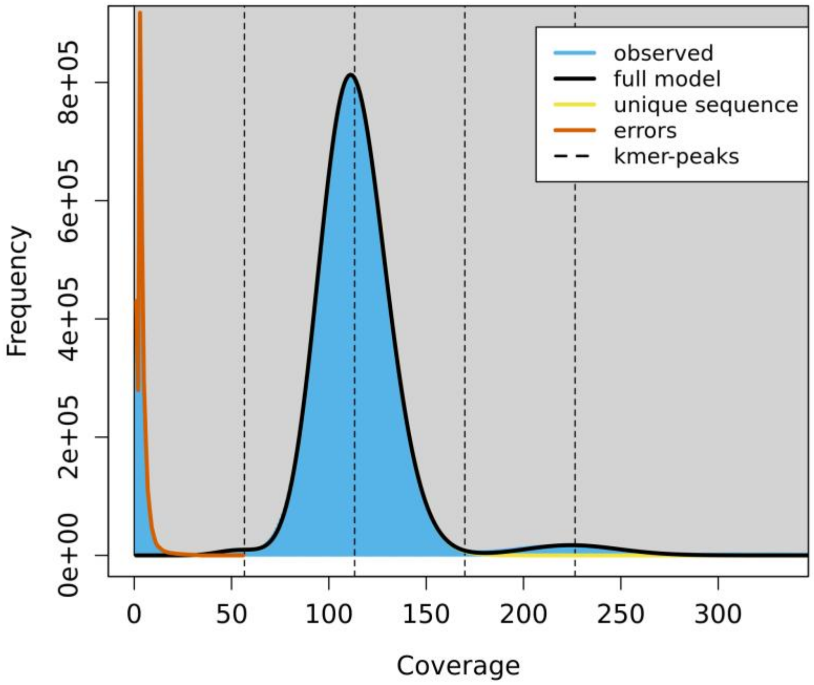

Figure S1. Histogram of the 19-mer depth distribution of the Illumina sequencing reads of *P. giganteus* plotted in GenomeScope. Blue areas indicate the observed k-mer frequencies and the black line indicates the fitted GenomeScope model (p = 2).

Figure S2

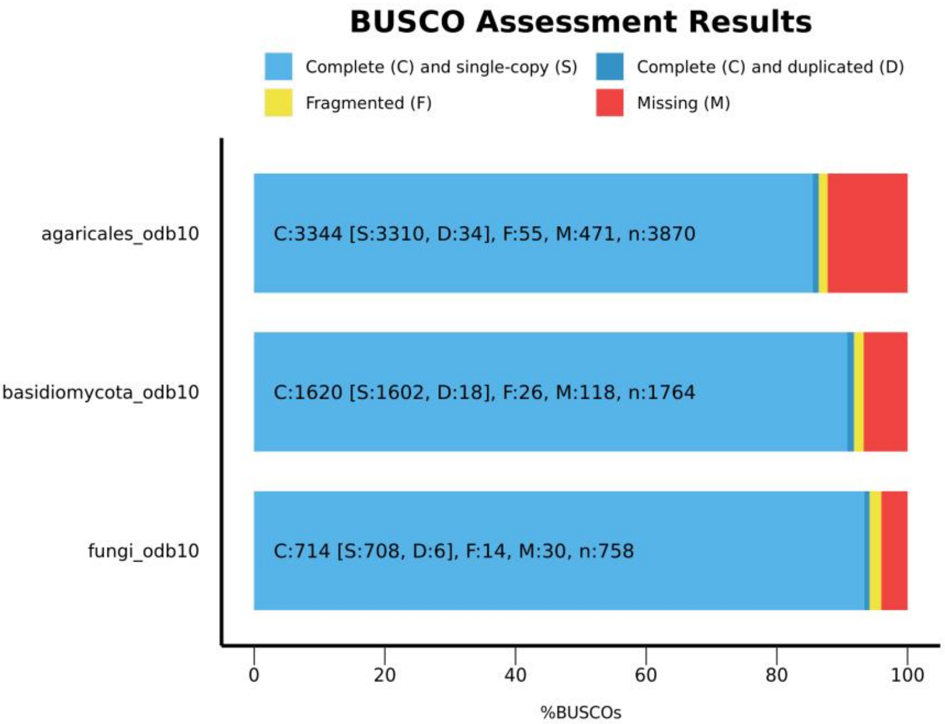

Figure S2. BUSCO assessment of the protein annotation completeness in P. giganteus with different BUSCO databases.

Figure S3

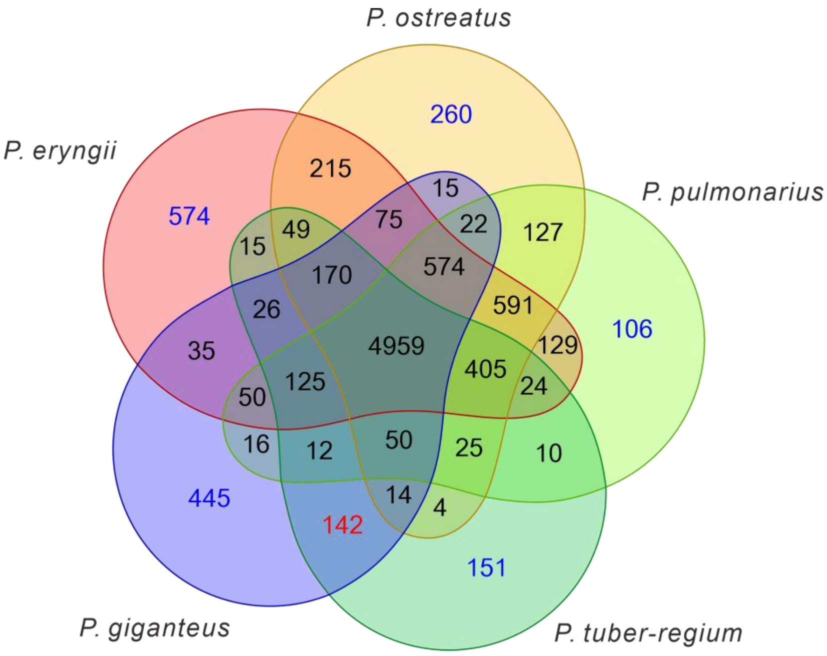

Figure S3. Venn diagram of the orthogroups of five *Pleurotus* species.

Table S1 Characteristics of the gene prediction of *P. giganteus*

Content	Number/length
Gene number	12628
Average gene length	1761.11 bp
Average protein length	586.04 aa
Total exon number	90508
Total exon/gene length	22239338 bp
Average exon length	245.72 bp
Average exon number per gene	7.17 bp
Total intron number	77880
Total intron length	23734624 bp
Average intron length	304.76 bp
Average intron number per gene	6.17 bp
